# Increased divergence but reduced variation on the Z chromosome relative to autosomes in *Ficedula* flycatchers: differential introgression or the faster-Z effect?

**DOI:** 10.1002/ece3.92

**Published:** 2012-02

**Authors:** Silje Hogner, Stein A Sæther, Thomas Borge, Torbjørn Bruvik, Arild Johnsen, Glenn-Peter Sætre

**Affiliations:** 1 National Centre for Biosystematics, Natural History Museum, University of OsloP.O. Box 1172, Blindern, NO-0318 Oslo, Norway; 2 Department of Biology, Centre for Ecological and Evolutionary Synthesis (CEES), University of OsloP. O. Box 1066, Blindern, N-0316 Oslo, Norway

**Keywords:** Allopatry, congeneric birds, faster-Z hypothesis, nuclear introns, Z chromosome polymorphism

## Abstract

Recent multilocus studies of congeneric birds have shown a pattern of elevated interspecific divergence on the Z chromosome compared to the autosomes. In contrast, intraspecifically, birds exhibit less polymorphism on the Z chromosome relative to the autosomes. We show that the four black-and-white *Ficedula* flycatcher species show greater genetic divergence on the Z chromosome than on the autosomes, and that the ratios of intraspecific polymorphism at Z-linked versus autosomal markers are below the neutral expectation of 75%. In all species pairs, we found more fixed substitutions and fewer shared polymorphisms on the Z chromosome than on the autosomes. Finally, using isolation with migration (IMa) models we estimated gene flow among the four closely related flycatcher species. The results suggest that different pattern of evolution of Z chromosomes and autosomes is best explained by the faster-Z hypothesis, since the estimated long-term gene flow parameters were close to zero in all comparisons.

## Introduction

When species diverge from each other, they are expected to gradually lose shared polymorphisms and accumulate fixed substitutions, due to random genetic drift and diversifying selection. Even though levels of polymorphism and divergence are expected to correlate across loci, divergence rates may differ between different parts of the genome. For example, loci under selection and linked sites will show different patterns of variation compared to those evolving neutrally (Nachman 1997; Wang et al. 1997; Fay and Wu 2000; Schlötterer 2003). Also demographic events can cause deviations from patterns expected under neutrality (Kreitman 2000). For example, a reduced population size would lead to a reduction of genetic variation, loss of alleles (especially rare ones), and random changes in allele frequencies (Frankham 1996). One useful approach for separating demographic processes and selection is to analyze patterns of divergence and polymorphism across several unlinked loci. Selection will typically only affect the target loci and closely linked regions whereas demographic processes will affect the whole genome.

Several studies have shown that the macro sex chromosome (X and Z in male and female heterogametic taxa, respectively) play an important role in the evolution of reproductive isolation between closely related species (Presgraves 2008; Qvarnström and Bailey 2009). Hybrids of the heterogametic sex typically suffer greater fitness reduction than homogametic hybrids (Haldane 1922), probably mainly because recessive alleles that are incompatible with heterospecific alleles at other loci get exposed to selection in the heterogametic sex but stay masked by dominance in the homogametic sex (Turelli and Orr 1995; Orr 1997). Also prezygotic barriers appear often to be controlled by sex-linked genes (reviewed by Qvarnström and Bailey 2009). For instance, studies of the pied flycatcher *Ficedula hypoleuca* and the collared flycatcher *F. albicollis* have shown that Z-linked genes control both male secondary sexual traits and female mate preferences (Sætre et al. 2003; Sæther et al. 2007).

The effective population size of Z-linked loci is ideally 0.75 of that of autosomal loci since females only have one Z-chromosome. Hence, at a balanced sex ratio and equal mutation rates the neutral expectation is that the nucleotide variation at Z-linked genes would be 3:4 of that at autosomal loci (Ellegren 2009). However, several studies of birds have reported much lower ratios than 0.75, suggesting that additional forces are reducing variation on the Z-chromosome relative to autosomes (Berlin and Ellegren 2004; Borge et al. 2005b; Storchova et al. 2010; Backström and Väli 2011; Elgvin et al. 2011). At the same time, a pattern of elevated interspecific divergence on the Z chromosome compared to the autosomes has also been found in birds (e.g., Borge et al., 2005b; Storchova et al. 2010; Elgvin et al. 2011). One explanation for this apparent nonneutral pattern is the faster-Z hypothesis (Charlesworth et al. 1987). Faster adaptive evolution on the Z is expected because (partially) recessive beneficial mutations are not masked by dominance in the heterogametic sex. Likewise, (partially) recessive deleterious mutations would be more effectively purged on the Z compared to autosomes due to hemizygous exposure. Associated selective sweeps on the Z chromosome would contribute to further reduce intraspecific polymorphism (Charlesworth et al. 1987; Borge et al. 2005b). Genetic drift can also contribute to a faster-Z effect because the lower effective population size of the Z chromosome would be associated with increased rates of genetic drift and thus an increased fixation rate of mildly deleterious mutations (Charlesworth et al. 1987; Mank et al. 2010).

A second hypothesis, here termed the differential introgression hypothesis, is that the accumulation of incompatibilities on the Z-chromosome may reduce the rate of introgression of Z-linked compared to autosomal genes and essentially produce the same pattern as predicted by the faster-Z hypothesis (Carling et al. 2010; Storchova et al. 2010; Backström and Väli 2011). The two hypotheses are not mutually exclusive, however. For instance, a faster-Z effect may speed up divergence and hence contribute to the accumulation of sex-linked incompatibilities that would reduce Z-linked introgression (e.g., Elgvin et al. 2011).

**Figure 1:**
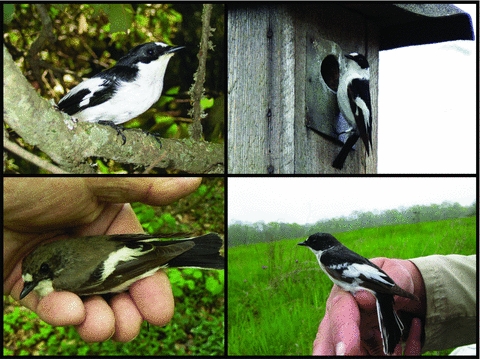
Males of the four study species. Top left: Atlas flycatcher *Ficedula speculigera* (photo: Gunilla Andersson), top right: collared flycatcher *F. albicollis* (photo: Miroslav Král), bottom left: pied flycatcher *F. hypoleuca* (photo: Miroslav Král), bottom right: semicollared flycatcher *F. semitorquata* (photo: Silje Hogner).

In this study, we test between the two hypotheses by analyzing pattern of polymorphism and divergence on Z-linked and autosomal loci in all the four species of the old world black-and-white flycatcher complex: the pied (*F. hypoleuca*), collared (*F. albicollis*), Atlas (*F. speculigera*), and semicollared flycatcher (*F. semitorquata*) (Fig. 1). The pied and collared flycatchers have earlier been investigated in some detail with respect to the role of selection in speciation (see Qvarnström et al. 2010; Sætre and Sæther 2010 for recent reviews). A moderate level of gene flow has been observed at autosomal loci between the collared and the pied flycatchers living in sympatry, while introgression on the Z chromosome is apparently absent (Sætre et al. 2003; Borge et al. 2005a; Backström et al. 2010). In a study of allopatric pied and collared flycatchers (from Spain and Italy, respectively), Borge et al. (2005b) reported significantly reduced levels of genetic variation but elevated rate of divergence at Z-linked loci in both species. However, Borge et al. (2005b) were unable to discriminate between the faster-Z effect and historical autosomal introgression as explanations for the pattern. At present, only the pied and the collared flycatcher have overlapping breeding ranges and hybridize. Even if some of the other species may also have hybridized in the past, we consider it unlikely that all the four flycatcher species have exchanged genes to the same extent. Hence, including more species is likely to help disentangling the relative importance of differential introgression and faster-Z in shaping the genomes of these birds.

To critically test between the faster-Z and the differential introgression hypotheses, we use isolation with migration model analysis (IMa) to estimate key demographic parameters, including effective population sizes, divergence times, and levels of gene flow between the species pairs at both the Z-linked and autosomal datasets. From the differential introgression hypothesis, we predict higher estimates of gene flow between the species at the autosomal compared to the Z-linked dataset, and that the amount of estimated gene flow on Z should be negatively associated with the degree of elevated divergence and reduced polymorphism on the Z relative to the autosomes among the different species pairs. From the faster-Z hypothesis, we predict no difference in amount of gene flow between autosomal and Z-linked loci.

## Materials and Methods

### Samples

**Figure 2:**
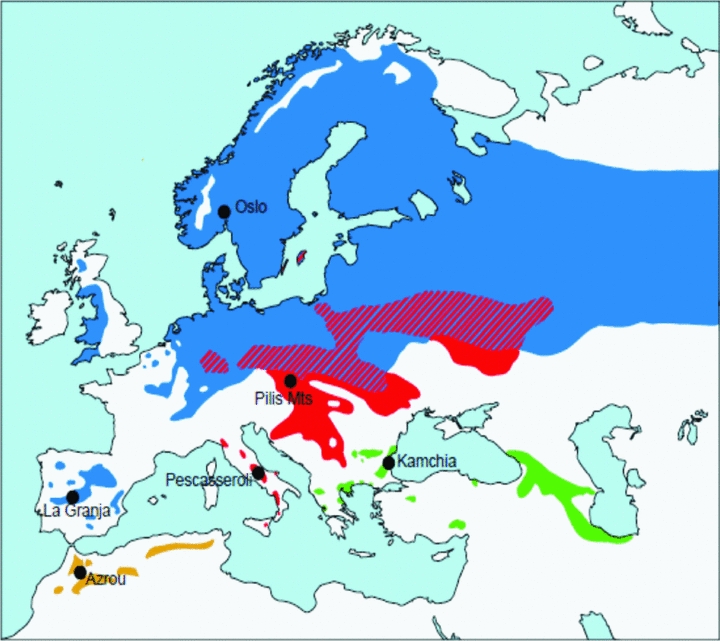
Breeding distribution of the Atlas flycatcher *Ficedula speculigera* (yellow), collared flycatcher *F. albicollis* (red), pied flycatcher *F. hypoleuca* (blue), and semicollared flycatcher *F semitorquata* (green). The hatched area indicates areas of distributional overlap between the pied and collared flycatcher. Sampling localities are indicated with black dots.

Adult breeding birds of the pied, collared, semicollared, and Atlas flycatchers were caught at their respective breeding grounds (Fig. 2). The Atlas flycatchers are from near Azrou, Morocco (*N* = 15 males, 33°26′N, 5°13′W), the collared flycatchers are from near Pescasseroli, Italy (*N* = 16 males, 41°48′N, 13°47′E) and from Pilis Mts, Hungary (*N* = 16 males, 47°43′N, 19°01′E), the pied flycatchers are from near Oslo, Norway (*N* = 16 males, 59°59′N, 10°46′E) and from La Granja, Spain (*N* = 14 males and two females, 40°14′N, 55°93′W), and the semicollared flycatchers are from Kamchia, Bulgaria (*N* = 12 males and three females, 42°53′N, 26°58′E). All birds were caught using mist nets and playback. Additionally, one male red-breasted flycatcher caught in Northern Moravia, Czech Republic, is included in the analysis to serve as an outgroup.

### DNA preparation

Approximately 25 µl of blood were collected from each individual by brachial venipuncture, and suspended in 1 mL Queen's lysis buffer (Seutin et al. 1991). DNA extractions were done using two different kits, QIAamp DNA Blood Mini Kit (Qiagen AB, Sollentuna, Sweden) and eZNA Blood DNA Kit (Omega Bio-Tek, Norcross, Georgia, USA), following the manufacturers protocols for each of the two kits.

Several primer pairs that have been tested on *Ficedula* flycatchers were available from previous studies (Primmer et al. 2002; Borge et al. 2005b). These primers were designed as described by Primmer et al. (2002), using chicken (*Gallus gallus*) sequences, available from GenBank, as templates. The primers were designed in exon sequences flanking introns of sizes appropriate for direct sequencing of both autosomal and Z-linked genes. New flycatcher-specific primers were designed for long introns when the amplification successes for these were variable (Borge et al. 2005b). In this study, we used primers that had high amplification success in earlier studies on the pied and collared flycatcher (Primmer et al. 2002; Sætre et al. 2003; Borge et al. 2005b) and that yielded high-quality sequences also in the other two species (Table 1).

**Table 1:** Primer information and amplification conditions

Locus	C^1^	Primer sequence (5′-3′)^2^	Annealing temperature
Acly-16	A	F: ACCATGAATTATCCCCAGGTGAG	55
		R: CAAAACCATTGGTACCCCACAG	
Alas1-8	A	F: CCGAGTCACATCATTCCCGT	55
		R: AGCAGCATCTGCAACCTGAA	
Fas-y	A	F: TGAAGAAGGTCTGGGTGGAGA	50
		R: CTCCAATAAGGTGCGGTGA	
Rho-1	A	F: CATCGAGGGCTTCTTTGCC	55
		R: TTTAGACACACAATTTCTATTTAACACCTGT	
Rpl30-3	A	F: CCAAGTTGGTCATCCTAGCCA	60
		R: GCCACTATAATGATGGACACCAGTC	
Tgfb2-5	A	F: TGCCTGCCATACATCCAGTG	55
		R: TGCTTGCTTCCTGAATGATCCT	
Aldob-6	Z	F: AGACCATGATCTCCAGCGCT	55
		R: CCTTCCAGGTAGACATGATG	
Brm-12	Z	F: CCCTATCTCATCATTGTTCC	50
		R: CACAGAAGGAGCCCATTTGT	
Chdz_15	Z	F: TAGAGAGATTGAGAACTACAGT	52
		R: GACATCCTGGCAGAGTATCT	
Chdz_18	Z	F: TACATACAGGCTCTACTCCT	58
		R: CCCCTTCAGGTTCTTTAAAA	
Ghr5_1	Z	F: GCTTCCATTATGTATCTTACC	55
		R: TTTGGCTTCTAGAGTTTTGCA	
GHR5_2	Z	F: ATGTTATTGCTTGTTCAGAGTG	58
		R: GAGTATTTGGAATAAAACAGCC	
Vldlr-8	Z	F: GTTATTGGCTATGAATGTGA	54
		R: GTTGATACAGATTTGGCTAC	
Vldlr-9	Z	F: AAGTGTGAATGTAGCCGTGG	54
		R: TCGGTTGGTGAAAATCAGAC	
Vldlr-12	Z	F: GTTCCTTCCTCATCCTCTTG	55
		R: ATAGACTGCCTCGTTCTCTC	

1 Chromosome class: A = autosomal; Z = Z-linked.

2 Borge et al. (2005).

The introns were amplified in PCR reaction volumes of 10 µl, containing dH_2_O, 1× PCR buffer II (Applied Biosystems), 1.5 mM magnesium, 0.2 mM dNTP (ABgene, Epsom, UK), 0.5 mM forward and reverse primer, 3% Dimethyl sulfoxide (DMSO), 0.25 U AmpliTaq DNA polymerase (Applied Biosystems, Foster City, California, USA), and approximately 50 ng DNA template. The amplifications were run on a DNA Engine Tetrad 2 (MJ Research, Waterton, MA). The following profile was used: 95°C for 1 min, 94°C for 30 sec, primer-specific annealing temperature (see Table 1) (55–60°C) for 30 sec, 72°C for 1 min, then the second and third step another 34–39 cycles before the last step, 72°C for 10 min. Three milliliters of PCR product was electrophoresed in 1% Tris/Borate/EDTA (TBE) agarose to confirm amplification success and to exclude any contamination.

The remaining PCR product was purified by digesting unincorporated nucleotides and primers using diluted (1:9) ExoSap-It (United States Biochemical Cleveland) run at 37°C for 45 min followed by 80°C for 15 min to inactivate the enzyme. The PCR products were then sequenced using BigDye Terminator sequencing buffer and v 3.1 Cycle Sequencing kit (Applied Biosystems). The sequences were aligned and edited using ClustalW in the program Mega 4.0.2 (Tamura et al. 2007) or in Sequencher 4.1 (Gene Codes, Ann Arbor, MI) and modified manually. Each base was called, using at least single-fold coverage sequencing reads for each strand. All sequences for each locus were adjusted to the same length as the shortest sequence of that locus for comparison. We analyzed intronic sequences from five Z-linked and six autosomal loci (accession numbers: JN995666-JN996468).

Previous reviewers have suggested that the sample sizes in this study are not sufficient to distinguish between the faster-Z and the differential introgression hypotheses. We disagree for three main reasons. First, previous studies utilizing a similar number of loci and individuals have found significant differences in level of polymorphisms and divergence between Z-linked and autosomal genes (e.g., Storchova et al. 2010; Elgvin et al. 2011). Second, our study is unique in the sense that we compare several geographically dispersed populations/species that differ in the likelihood of having experienced episodes of introgressive hybridization. Although both hypotheses predict reduced polymorphism and increased divergence of Z-linked compared to autosomal loci, they differ with respect to the heterogeneity of this signal. The faster-Z hypothesis predicts that all pairs of populations will be similarly affected, whereas the differential introgression hypothesis would only affect population pairs that have hybridized extensively after partial reproductive isolation has developed. Finally, our sample sizes allow good estimates of historic gene flow between several population pairs using a coalescent simulation framework (see below).

### Cloning

Sequences with difficult gaps were cloned in order to get the respective haplotypes. All cloning was performed using the Zero Blunt TOPO PCR cloning kit (Invitrogen) and transformed into *Escherichia coli* DH5α chemically competent cells as recommended by Invitrogen (Invitrogen, Carlsbad, California, USA). The transformed cells were then grown on LB agar containing Kanamycin (100 µg/mL) as a selection marker. Eight positive colonies from each cloned individual were picked with sterile toothpicks and diluted with 6 µl dH_2_O and used directly as PCR template. Amplification and purification was performed as described for genomic DNA and standard M13 primers were used.

### Polymorphism and divergence

The program DnaSP 4.0 (Librado and Rozas 2009) was used to analyze polymorphism. Two common measures of nucleotide polymorphism were calculated: π, the average number of nucleotide differences per site between two sequences (Nei 1987) and θ_W_, the population mutation parameter estimated from the number of segregating sites in the aligned sample of sequences. θ = 4N_e_µ for autosomal loci and 3N_e_µ for Z-linked loci, in which N_e_ is the effective population size and µ is the neutral mutation rate (Nei 1987). The π and θ_W_ parameters were estimated with standard deviations in DnaSP.

We calculated the Z:autosomal ratio (Z_θ_:A_θ_) of average pairwise sequence differences. This was done by dividing the average pairwise sequence difference per nucleotide for each Z-linked locus by the average pairwise sequence difference per nucleotide for all autosomal loci.

Polymorphisms were divided into four categories for each of the species pairs: variable sites exclusive to one of the species, shared polymorphisms, fixed differences, and the average number of pairwise differences.

To test if the levels of polymorphism and divergence were correlated between loci and species as predicted under neutrality, multilocus Hudson–Kreitmann–Aguade (HKA) tests (Hudson et al. 1987) were run for all species pairs for both Z-linked and autosomal loci using the online software of Jody Hey's lab (http://lifesci.rutgers.edu/∼heylab). Deviations from the expected relationship may indicate that selection has affected genetic variation. The polymorphism and divergence data from Table 2 and Table S1 were used for these tests. For all species pairs, 10,000 coalescent simulations were run to assess significance.

**Table 2:** Polymorphism summaries of Z-linked genes in the four black-and-white *Ficedula* flycatcher species

Gene	Species^1^	N^2^	L^3^	K^4^	S^5^	s^6^	Π^7^	θw^8^	Tajima's D^9^
Aldob-6	A-Mar	30	437	10	0	0	0	0	–
	C-It	30	437	9	1	0	0.0004	0.0006	–0.409
	P-Spa	31	437	9	3	1	0.0006	0.0017	0.179
	C-Hun	32	437	9	1	0	0.0004	0.0006	–0.448
	P-Nor	32	437	9	0	0	0	0	–
	S-Bul	27	437	10	1	1	0.0002	0.0006	−1.154
Brm-12	A-Mar	28	1439	30	20	11	0.0017	0.0036	−1.897
	C-It	28	1438	33	11	4	0.0014	0.0002	−0.910
	P-Spa	24	1439	34	12	1	0.0030	0.0022	1.118
	C-Hun	30	1439	30	26	11	0.0037	0.0046	−0.704
	P-Nor	26	1438	32	23	8	0.0033	0.0042	−0.773
	S-Bul	25	1434	23	15	8	0.0030	0.0043	−1.077
Chdz	A-Mar	30	638	14	1	0	0.0005	0.0004	0.216
	C-It	29	638	13	1	1	0.0001	0.0004	−1.149
	P-Spa	31	638	14	3	1	0.0006	0.0012	−1.183
	C-Hun	30	638	13	4	2	0.0006	0.0016	−1.574
	P-Nor	32	638	14	1	0	0.0004	0.0004	0.147
	S-Bul	27	638	13	1	0	0.0004	0.0004	0.017
Ghr	A-Mar	30	548	9	3	0	0.0021	0.0014	1.147
	C-It	30	555	9	4	3	0.0010	0.0018	−0.796
	P-Spa	31	555	10	4	1	0.0008	0.0018	−1.430
	C-Hun	32	555	9	4	0	0.0014	0.0018	−0.541
	P-Nor	32	555	10	6	2	0.0013	0.0027	−1.456
	S-Bul	27	555	10	5	2	0.0020	0.0023	−0.452
Vldlr	A-Mar	30	562	5	9	2	0.0030	0.0040	−0.771
	C-It	30	562	4	12	3	0.0062	0.0054	0.464
	P-Spa	28	562	4	3	0	0.0007	0.0014	−1.115
	C-Hun	32	558	4	13	6	0.0049	0.0058	−0.481
	P-Nor	30	562	4	4	2	0.0011	0.0018	−0.960
	S-Bul	27	562	4	11	8	0.0026	0.0051	−1.598
**Total Z**	**A-Mar**	**30**	**3624**	**68**	**33**	**13**	**0.0014**	**0.0015**	**−0.161**
	**C-It**	**30**	**3631**	**68**	**29**	**11**	**0.0020**	**0.0021**	**−0.102**
	**P-Spa**	**31**	**3631**	**71**	**25**	**4**	**0.0010**	**0.0015**	**−1.113**
	**C-Hun**	**32**	**3641**	**65**	**48**	**19**	**0.0024**	**0.0029**	**−0.580**
	**P-Nor**	**32**	**3641**	**69**	**34**	**12**	**0.0009**	**0.0012**	**−0.736**
	**S-Bul**	**27**	**3626**	**60**	**33**	**19**	**0.0013**	**0.0021**	**−1.338**
Acly-16	A-Mar	30	358	5	0	0	0	0	–
	C-It	30	358	5	1	1	0.0002	0.0007	−1.147
	P-Spa	30	358	5	2	1	0.0006	0.0014	−1.256
	C-Hun	32	358	5	2	2	0.0004	0.0014	−1.504
	P-Nor	32	358	5	0	0	0	0	–
	S-Bul	30	358	5	1	1	0.0002	0.0007	−1.147
Alas1-8	A-Mar	30	290	12	7	2	0.0051	0.0061	−0.479
	C-It	30	290	14	9	4	0.0054	0.0078	−0.957
	P-Spa	34	290	15	6	2	0.0027	0.0051	−1.302
	C-Hun	32	290	13	9	3	0.0062	0.0077	−0.620
	P-Nor	32	290	15	7	3	0.0023	0.0060	−1.786
	S-Bul	30	288	13	9	2	0.0083	0.0079	0.144
Fas-y	A-Mar	26	551	10	0	0	0	0	–
	C-It	26	552	11	7	3	0.0018	0.0033	−1.377
	P-Spa	34	551	10	1	1	0.0001	0.0004	−1.138
	C-Hun	32	550	11	1	1	0.0001	0.0005	−1.142
	P-Nor	30	551	10	2	1	0.0009	0.0009	−0.136
	S-Bul	30	551	9	6	1	0.0013	0.0028	−1.515
Rho-1	A-Mar	30	372	8	8	2	0.0049	0.0054	−0.306
	C-It	30	372	7	6	1	0.0049	0.0041	0.577
	P-Spa	30	372	7	8	1	0.0072	0.0054	0.970
	C-Hun	26	371	7	11	6	0.0060	0.0078	−1.002
	P-Nor	32	372	7	6	1	0.0057	0.0047	0.644
	S-Bul	30	372	6	12	4	0.0070	0.0081	−0.450
Rpl30-3	A-Mar	30	983	17	22	7	0.0058	0.0057	0.107
	C-It	30	983	16	21	2	0.0053	0.0054	−0.051
	P-Spa	30	983	17	14	7	0.0021	0.0036	−1.392
	C-Hun	32	982	20	23	13	0.0043	0.0058	−0.893
	P-Nor	28	983	17	21	2	0.0083	0.0055	1.806
	S-Bul	26	983	21	15	3	0.0055	0.0040	1.295
Tgfb2-5	A-Mar	30	402	5	6	2	0.0042	0.0038	0.308
	C-It	30	402	5	6	4	0.0021	0.0038	−1.277
	P-Spa	34	402	5	3	0	0.0021	0.0018	0.289
	C-Hun	32	402	5	11	4	0.0047	0.0068	−1.354
	P-Nor	32	402	5	7	4	0.0030	0.0043	−0.875
	S-Bul	30	401	5	6	3	0.0043	0.0038	−0.075
**Total A**	**A-Mar**	**30**	**2956**	**57**	**43**	**13**	**0.0045**	**0.0045**	**−0.054**
	**C-It**	**30**	**2957**	**58**	**50**	**15**	**0.0040**	**0.0045**	**−0.455**
	**P-Spa**	**34**	**2956**	**59**	**34**	**12**	**0.0013**	**0.0020**	**−0.986**
	**C-Hun**	**32**	**2958**	**61**	**57**	**29**	**0.0031**	**0.0044**	**−1.177**
	**P-Nor**	**32**	**2975**	**59**	**43**	**11**	**0.0028**	**0.0037**	**−0.799**
	**S-Bul**	**30**	**2956**	**59**	**49**	**14**	**0.0038**	**0.0044**	**−0.557**

1 A-Mar = Atlas flycatcher from Morocco; C-It = collared flycatcher from Italy; P-Spa = pied flycatcher from Spain; C-Hun = collared flycatcher from Hungary; P-Nor = pied flycatcher from Norway; S-Bul = semicollared flycatcher from Bulgaria.

2 Number of sites surveyed.

3 Sequence length.

4 Number of divergent sites with outgroup.

5 Number of segregating sites.

6 Number of singleton sites.

7 Average pairwise sequence difference per nucleotide (Nei 1987).

8 Expected heterozygosity per nucleotide (Watterson 1975).

9 None of the D-values significant after correcting for multiple tests.

DnaSP was used to compute Tajima's *D* (Tajima 1989). This neutrality test is based on the allele frequency spectrum. It can be used to infer previous evolutionary and demographic events that the populations have experienced. Negative values of Tajima's *D* reflect an excess of rare polymorphisms in the populations, while positive values indicates an excess of intermediate-frequency alleles. An excess of rare alleles might result from positive selection or an increase in population size, whereas an excess of intermediate-frequency alleles might result from balancing selection or a population bottleneck (Akey et al. 2004).

### Population divergence

For sequences that contained more than one heterozygous site, and in which cloning was not performed, haplotypes were inferred using the programme Phase version 2.1.1 (Stephens and Donnelly 2003). Each run had the following values set, iterations (10,000), thinning interval (1), and burn-in (1000). Harrigan et al. (2008) showed that haplotypes with a PHASE probability greater than 0.5 are reliable. Consequently, we used 0.5 as a lower limit.

Analysis of molecular variance (AMOVA) was run using Arlequin v 3.5 (Excoffier et al. 2005) to examine the genetic structure of the populations. The variance was partitioned into four: between the species, between individuals within the species, among individuals within populations, and within the different individuals. Pairwise species differentiation was estimated using *F*_ST_ (Weir and Cockerham 1984), with default settings in the population comparisons. These *F*_ST_ values can be used as short-term genetic distances between populations. The null distribution of pairwise *F*_ST_ values is obtained by permutating haplotypes between populations under the hypothesis of no differences between populations. The *P*-value of the test is given as the proportions of simulations giving an *F*_ST_ value larger or equal to the observed one.

### IMa model

Recombination was calculated as the minimum number of recombination events (RM) using the four-gamete test in DnaSP (Hudson and Kaplan 1985). The sites with recombination were excluded from the coalescent analysis.

In order to estimate the timing, magnitude of divergence and gene flow between the four species, a coalescent framework, the IMa (Nielsen and Wakeley 2001; Hey and Nielsen 2007) model was used. We included one population from each of the four species. We chose the Norwegian population of the pied flycatcher because previous analyses had indicated inbreeding effects (violations of the neutral model) in the alternative Spanish population (Haavie et al. 2000). Further, we included the Italian population of the collared flycatcher because the alternative Hungarian population is located close to the pied × collared flycatcher hybrid zone, and hence, could have been affected by recent introgression (Borge et al. 2005a).

Initial IMa runs were conducted using wide priors set as recommended in the IMa manual. Two final sets of runs were conducted, one with only one chain (run multiple times with different random seed numbers) and one with 10 chains and 10 swapping events. First, the multiple final runs were conducted with a length between 8.0 × 10^7^ and 9.0 × 10^8^ generations, where the first 30% were discarded as burn-in. Second, a run contained 10 chains and 10 chain swap attempts per step, with a burn-in period of 10–15% and a run length between 2.0 × 10^7^ and 3.0 × 10^7^ generations. No runs were stopped before the effective sample size values had exceeded at least 200. Two separate IMa analyses were run for each species pairs: (1) the autosomal dataset containing six autosomal loci and (2) the Z-linked dataset containing five Z-linked loci. Two independent runs, with different random seed numbers, were conducted per comparison.

In order to convert the parameter estimates into demographic quantities, we used a neutral mutation rate of 1.35 × 10^–9^ substitutions per site per year for autosomal genes (Ellegren 2007) and 1.45 × 10^–9^ substitutions per site per year for Z-linked genes (Axelsson et al. 2004; Ellegren 2007). Using these mutation rates and setting the generation time to one year, we calculated the geometric mean of mutation rates per locus for the different datasets. There was good agreement between the independent runs and therefore we only report the results from the longest independent run.

## Results

### Intraspecific polymorphism

**Figure 3:**
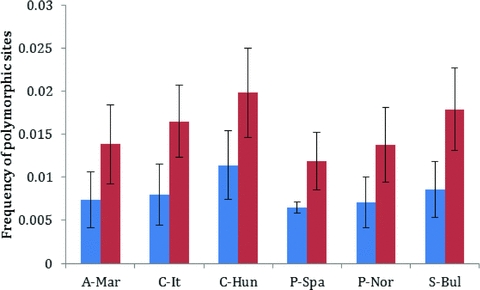
Mean frequency (± SE) of polymorphic sites for Z-linked (blue) and autosomal (red) loci in the six populations. A-Mar, Atlas flycatcher from Morocco; C-It, collared flycatcher from Italy; C-Hun, collared flycatcher from Hungary; P-Spa, pied flycatcher from Spain; P-Nor, pied flycatcher from Norway; S-Bul, semicollared flycatcher from Bulgaria.

**Figure 4:**
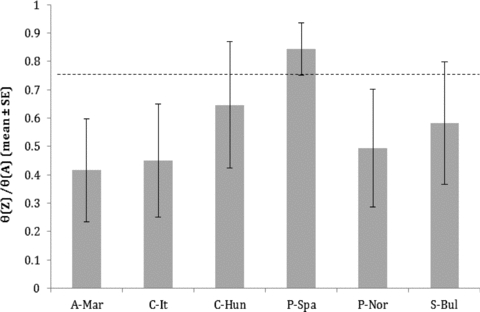
The ratio of Z to autosomal variation measured as the expected heterozygosity for each Z-linked locus (θ(z)) divided by the expected heterozygosity for all autosomal loci (θ(A)). The dashed line indicates the expected value of 0.75. A-Mar, Atlas flycatcher from Morocco; C-It, collared flycatcher from Italy; C-Hun, collared flycatcher from Hungary; P-Spa, pied flycatcher from Spain; P-Nor, pied flycatcher from Norway; S-Bul, semicollared flycatcher from Bulgaria.

We found that the frequency of polymorphic sites was quite heterogeneous among loci and somewhat higher overall for the autosomal loci than for the Z-linked loci (Table 2; Fig. 3). The total frequency of polymorphic sites was significantly higher at the autosomal loci than the Z-linked ones in the Atlas flycatcher (*P* = 0.048), the Italian collared flycatcher (*P* = 0.0013), The Spanish pied flycatcher (*P* = 0.050), and the semicollared flycatcher (*P* = 0.0072), and nearly significantly higher also in the Hungarian collared flycatcher (*P* = 0.060) and the Norwegian pied flycatcher (*P* = 0.064) (Fisher's exact tests). Combining the *P*-values above in a Fisher's combined probability test yields a highly significant test statistic (χ^2^ = 46.35, df = 12, *P* < 0.0001). Hence, there is an overall reduction in the frequency of polymorphic sites at the Z-linked loci in the dataset. For all species except the Spanish pied flycatchers, the Z_θ_:A_θ_ ratios were below the expected value of 0.75 (Fig. 4). The ratio was significantly lower than 0.75 in the Atlas flycatcher (*t* = 2.72, df = 10, *P* = 0.022, one-sample *t*-test) and the Italian collared flycatcher (*t* = 2.23, df = 10, *P* = 0.050) and nearly so also in the Norwegian pied flycatcher (*t* = 1.82, df = 10, *P* = 0.098). The other ratios did not differ significantly from the expectation of 0.75 (*P* > 0.1, one-sample *t*-tests). However, when combining all Z_θ_:A_θ_ ratios, Fisher's combined *P*-values were significant (χ^2^ = 25.80, df = 12, *P* = 0.012).

### Fixed and shared polymorphisms between species

**Figure 5:**
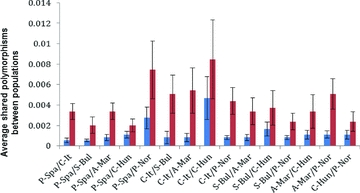
Average number of shared polymorphisms ± SE at Z-linked (blue) and autosomal (red) loci between the different flycatcher populations. A-Mar, Atlas flycatcher from Morocco; C-It, collared flycatcher from Italy; C-Hun, collared flycatcher from Hungary; P-Spa, pied flycatcher from Spain; P-Nor, pied flycatcher from Norway; S-Bul, semicollared flycatcher from Bulgaria.

**Figure 6:**
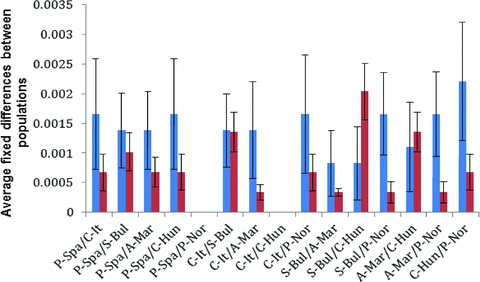
Average number of fixed differences ± SE at Z-linked (blue) and autosomal (red) loci between the different flycatcher populations. A-Mar, Atlas flycatcher from Morocco; C-It, collared flycatcher from Italy; C-Hun, collared flycatcher from Hungary; P-Spa, pied flycatcher from Spain; P-Nor, pied flycatcher from Norway; S-Bul, semicollared flycatcher from Bulgaria.

For all the species combinations, the average number of shared polymorphisms was higher at the autosomal than the Z-linked loci (Fig. 5). In contrast, the level of fixed differences was in most cases higher at the Z-linked than at the autosomal loci (Fig. 6). The ratio of fixed differences to shared polymorphisms was significantly higher at the Z-linked loci compared to autosomal ones in five of 13 species comparisons (Atlas flycatcher vs. Italian collared flycatcher, Atlas flycatcher vs. Norwegian pied flycatcher, Italian collared flycatcher vs. Norwegian pied flycatcher, Italian collared flycatcher vs. Spanish pied flycatcher, and semicollared flycatcher vs. Norwegian pied flycatcher) (Fisher's exact tests: *P* < 0.05); and nearly significantly higher also between Atlas flycatcher versus Spanish pied flycatcher, Hungarian collared flycatcher versus Norwegian pied flycatcher, and Atlas flycatcher versus semicollared flycatcher (Fisher's exact tests: 0.1 > *P* > 0.05). However, when using Bonferroni correction for multiple comparisons and setting alpha to 0.05, only three comparisons remain statistically significant (Atlas flycatcher vs. Italian collared flycatcher, Italian collared flycatcher vs. Norwegian pied flycatcher, and Atlas flycatcher vs. Norwegian pied flycatcher).

### Tests of neutrality

HKA tests for all population comparisons revealed no significant deviations from expected values at neither the autosomal nor the Z-linked loci (all *P*-values > 0.17; Table S2). We also performed neutrality test based on the allele frequency distribution, namely Tajima's *D* for each locus. Again, none of the tests showed any significant deviations from neutrality (Table 2). For all species except the Atlas flycatcher and the Norwegian pied flycatchers, the Tajima's *D* values were slightly negative at both the Z-linked and the autosomal loci. Hence, the allele frequency spectra closely match the neutral expectations, with a small skew toward rare alleles (and a small skew toward alleles of intermediate frequencies in the Atlas flycatcher and the Norwegian pied flycatchers).

### Population divergence

AMOVA for all Z-linked loci combined showed that about 76% of the variation could be explained by differences between species, 7% by variation among individuals within populations, 16% by variation within individuals, and less than 1% from variation among populations within groups. For all autosomal loci combined, 47% of the variation could be explained by differences between species, 5% by variation between individuals within populations, 47% by variation within individuals, and around 1% by variation among populations within groups (Table 3). We also computed *F*_ST_ values for each of the species pairs (Table 4). All the *F*_ST_ values were high, quite similar between the different species pairs and significantly larger than zero, all *P* < 0.01. Hence, all the four species are genetically strongly differentiated from each other. Using pairwise comparisons, *F*_ST_ was significantly higher at the Z-linked dataset compared to the autosomal dataset (Table 2; *t* = 7.06, df = 14, *P* < 0.0001, paired *t*-test). Restricting the test to between-species comparisons further increased the significance (*t* = 10.1, df = 12, *P* < 0.000001, paired *t*-test).

**Table 3:** Analysis of molecular variation (AMOVA) between six populations (four species) of *Ficedula* flycatchers

Source of variation	df	Sum of squares	Variance components	Percentage of variation	*P* -value
Z-linked loci					
Between species	3	1380.82	5.38	76.41	<0.05
Among populations within species	2	8.77	0.04	0.53	<0.05
Among individuals within populations	170	361.68	0.50	7.16	<0.001
Within individuals	176	197.00	1.12	15.90	<0.001
Total	351	1928.26	7.04		
Autosomal loci					
Between species	3	643.12	2.28	47.09	<0.05
Among populations within species	2	12.45	0.05	1.11	<0.01
Among individuals within populations	182	503.96	0.26	5.30	<0.001
Within individuals	188	424.00	2.26	46.50	<0.001
Total	375	1574.53	4.85		

**Table 4:** Pairwise *F*_ST_ values between six populations (four species) of *Ficedula* flycatchers; Atlas flycatcher from Morocco (A-Mar), collared flycatcher from Italy (C-It) and Hungary (C-Hun), pied flycatcher from Spain (P-Spa) and Norway (P-Nor), and semicollared flycatcher from Bulgaria (S-Bul). All values are significant except the one marked with an asterisk

	P-Spa	P-Nor	C-Hun	C-It	S-Bul	A-Mar
Z-linked loci						
P-Spa	0.000					
P-Nor	0.049	0.000				
C-Hun	0.692	0.699	0.000			
C-It	0.703	0.710	0.016	0.000		
S-Bul	0.760	0.763	0.557	0.578	0.000	
A-Mar	0.851	0.853	0.819	0.827	0.853	0.000
Autosomal loci						
P-Spa	0.000					
P-Nor	0.034	0.000				
C-Hun	0.420	0.438	0.000			
C-It	0.484	0.504	0.020^*^	0.000		
S-Bul	0.466	0.498	0.412	0.441	0.000	
A-Mar	0.605	0.629	0.374	0.382	0.500	0.000

### IMa analysis

**Figure 7:**
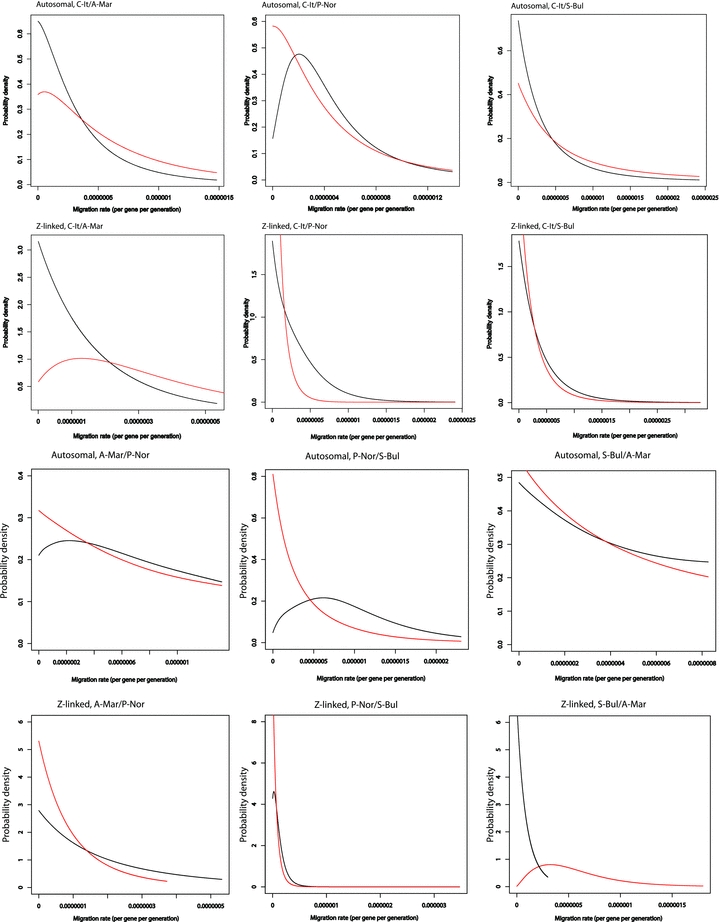
Posterior probability distribution of the migration rate per generation for autosomal and Z-linked loci, respectively. Only values from the run with 10 chains and chain swapping are shown here. A-Mar, Atlas flycatcher from Morocco; C-It, collared flycatcher from Italy; P-Nor, pied flycatcher from Norway; S-Bul, semicollared flycatcher from Bulgaria. Black lines: M1, migration rate from population 2 to 1. Red lines: M2, migration rate from population 1 to 2.

**Figure 8:**
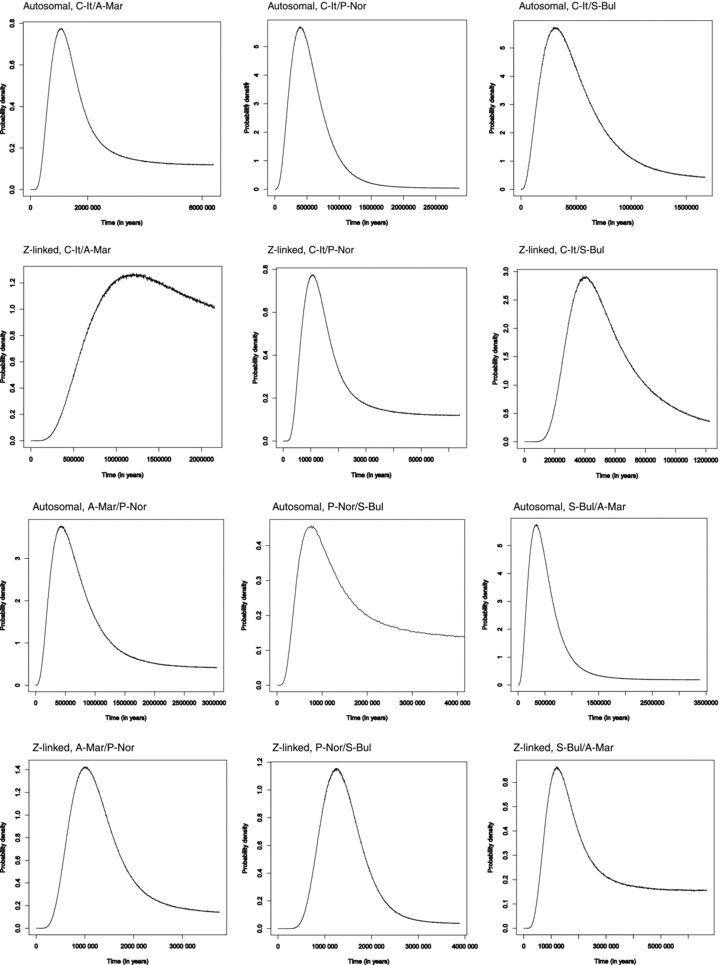
Posterior probability distribution of the time since divergence (in years) for autosomal and Z-linked loci, respectively. Only values from the run with 10 chains and chain swapping are shown here. A-Mar, Atlas flycatcher from Morocco; C-It, collared flycatcher from Italy; P-Nor, pied flycatcher from Norway; S-Bul, semicollared flycatcher from Bulgaria.

**Figure 9:**
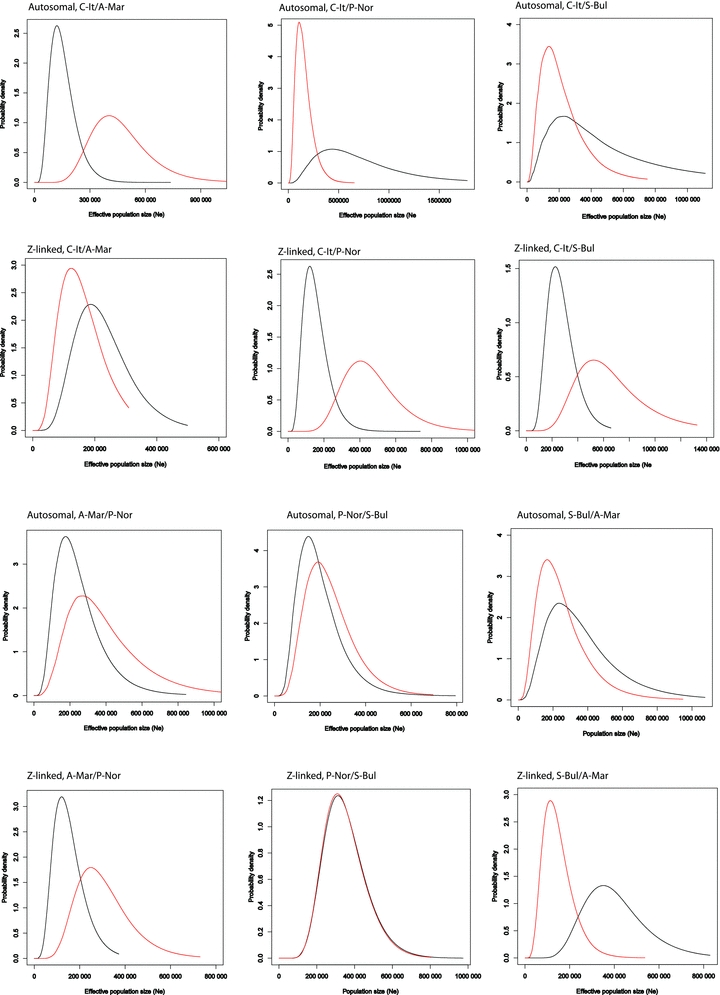
Posterior probability distribution of the effective population size for autosomal and Z-linked loci, respectively. Only values from the run with 10 chains and chain swapping are shown here. A-Mar, Atlas flycatcher from Morocco; C-It, collared flycatcher from Italy; C-Hun, collared flycatcher from Hungary; P-Spa, pied flycatcher from Spain; P-Nor, pied flycatcher from Norway; S-Bul, semicollared flycatcher from Bulgaria. Black lines: Q1, effective population size for population 1. Red lines: Q2, effective population size for population 2.

We found very low estimates of levels of gene flow at both the Z-linked and autosomal dataset in all species pairs (Fig. 7; Table 5). In fact, the posterior probability densities approached zero migration in all runs (Fig. 7). The divergence time estimates suggested more recent splits between the species pairs at the autosomal dataset (range 298,086–756,159 years ago) compared to the Z-linked dataset (range 385,550–1, 245,572 years ago) (Fig. 8; Table 5). The effective population size estimates were high in all four species and quite similar at the autosomal and Z-linked datasets (Fig. 9; Table 5). For most parameters in most runs, we got good convergence and the posterior probability tails were zero or close to zero within the parameter range. For the ones that did not reach zero, the highest and most probable peak was within the prior parameter range.

**Table 5:** Summary of the posterior probability values from IMa-analyses scaled by per gene per generation mutation rate (see methods). The highest point estimate for each parameter is given plus the lower and higher boundaries for the 90% highest posterior density. Only values from the run with 10 chains and chain swapping are given

Species pair		T^1^	Q1^2^	Q2^3^	M1^4^	M2^5^
A-Mar/P-Nor		430,882	176,067	269,301	2.2	0.067
	A	(105,120–2,227,958)	(64,632–435,339)	(97,691–691,454)	(0.067–114.3)	(0.067–113.0)
		993,988	120,490	248,825	0.027	0.02
	Z	(403,983–2,711,389)	(51,954–251,644)	(123,872–483,418)	(0.027–39.1)	(0.02–22.3)
S-Bul/A-Mar		333,689	234,937	166,468	0.043	0.04
	A	(52,668–1,623,304)	(76,838–657,504)	(51,727–454,920)	(0.043–70.0)	(0.043–70.1)
		1,205,473	351,081	113,618	0.018	32.3
	Z	(530,942–5,800,298)	(189,419–613,046)	(48,085–246,354)	(0.018–19.4)	(3.8–106.5)
C-It/S-Bul		298,086	231,825	136,164	0.12	0.12
	A	(72,478–1,097,840)	(51,694–838,924)	(31,887–416,841)	(0.12–118.7)	(0.1–163.5)
		385,550	225,718	523,381	0.16	0.16
	Z	(201,676–964,166)	(107,441–430,788)	(265,995–1,030,164)	(0.16–96.5)	(0.16–70.9)
C-It/A-Mar		526,701	324,531	111,379	0.074	5.4
	A	(180,796–1,682,073)	(130,989–778,973)	(41,767–285,313)	(0.074–81.6)	(0.074–107.6)
		119,6157	186,969	124,053	0.027	13.0
	Z	(681,592–2,152,259)	(88,101–362,236)	(55,285–255,409)	(0.027–35.5)	(0.080–78.1)
P-Nor/S-Bul		756,159	148,231	191,783	6.2	0.12
	A	(300,063–2,399,3049)	(57,612–338,128)	(81,102–402,084)	(2.2–17.0)	(0.12–10.8)
		1,245,572	311,134	309,255	2.3	0.17
	Z	(627,644–2,310,430)	(172,845–529,287)	(173,330–519,960)	(0.17–2.7)	(0.17–1.7)
C-It/P-Nor		397,431	435,041	106,529	2.0	0.069
	A	(116,150–1,045,707)	(123,672–1,251,683)	(36,384–278,219)	(0.070–8.8)	(0.069–9.0)
		1,096,693	121,179	403,247	1.2	0.12
	Z	(393,300–5,233,845)	(51,195–257,437)	(219,780–707,774)	(1.2–7.8)	(0.12–2.3)

A-Mar = Atlas flycatcher from Morocco; C-it = collared flycatcher from Italy; P-Nor = pied flycatcher from Norway; S-Bul = semicollared flycatcher from Bulgaria.

1 Time since divergence.

2 Effective population size 1.

3 Effective population size 2.

4 Migration rate from population 2 to 1 (per gene per generation, ×10^–8^).

5 Migration rate from population 1 to 2 (per gene per generation, ×10^–8^).

## Discussion

We analyzed polymorphism and divergence at five Z-linked and six autosomal loci in and between the four black-and-white *Ficedula* flycatcher species. The Z-linked markers exhibited reduced levels of polymorphism, yet elevated levels of differentiation between the species pairs compared to the autosomal ones. Such a pattern of elevated divergence and reduced polymorphism has previously been reported also among other closely related bird species (Berlin and Ellegren 2004; Borge et al. 2005b; Storchova et al. 2010; Backström and Väli 2011; Elgvin et al. 2011), and has alternately been attributed to faster adaptive divergence on the Z chromosome (the faster-Z hypothesis) and/or reduced introgression on the Z chromosome due to accumulation of sex-linked incompatibilities (the differential introgression hypothesis). Below, we discuss our results in more detail to try to disentangle which of the hypotheses best explains the observed patterns.

### Comparison of species pairs

Of the four focal taxa, only the pied flycatcher and collared flycatcher have overlapping breeding ranges at present (Fig. 2). In sympatric populations of these two species, some hybridization occurs (Qvarnström et al. 2010; Sætre and Sæther 2010). Evidence suggests that some autosomal introgression may occur in these sympatric populations whereas Z-linked introgression is apparently absent (Borge et al. 2005a). This result is consistent with the differential introgression hypothesis. However, heterospecific autosomal alleles have only been found within the hybrid zones and not in adjacent allopatric populations (Borge et al. 2005a). We thus consider it likely that the individuals inferred to possess introgressed alleles in Borge et al. (2005a) are actually recent backcrosses. Wiley et al. (2009) demonstrated that hybrid problems (low fertility) is not restricted to F1-hybrids but also occurs in first- and second-generation backcrosses (and possibly beyond). Hence, current introgression appears to be too low to significantly affect genetic variation of the pied and collared flycatcher except in the very heart of their hybrid zones.

Species distributions are not static and it is certainly possible that some of the species pairs have experienced secondary contact in the past, following their initial split. However, the pattern of elevated divergence and reduced polymorphism at the Z-linked loci appeared rather consistent among the different pairs of species: The Z_θ_:A_θ_ ratio was below the expected ratio of 0.75 in all populations except the Spanish pied flycatcher. However, in a previous study where a larger number of Z-linked and autosomal markers were analyzed, a significantly reduced Z_θ_:A_θ_ ratio was reported also in the latter population (Borge et al. 2005b), suggesting that the apparent heterogeneity in the present study may be mainly due to the lower number of loci included. On the other hand, species differentiation was consistently larger at the Z-linked compared to the autosomal loci. We would think that the degree of historic introgression and the degree to which such introgression would have been biased toward the autosomes are likely to have varied among the species pairs. Accordingly, we would expect the pattern of polymorphism and divergence to have been more heterogeneous than what we observe if differential introgression was the major factor affecting Z-linked and autosomal loci differentially.

### IMa analysis

According to the IMa analyses, our estimates of gene flow between the species pairs do not support the differential introgression hypothesis. We found no evidence for elevated rates of autosomal gene flow among any of the species pairs. The estimates of historic gene flow were close to zero for all species pairs at both the Z-linked and the autosomal dataset. Rather, our analyses are consistent with a scenario of classical allopatric speciation. The four focal taxa are well differentiated according to both the autosomal and the Z-linked dataset, having diverged several hundred thousand years ago or even more than a million years ago according to some of the estimates, not very different from previous estimates based on divergence at mitochondrial DNA (e.g., Sætre et al. 2001).

### Faster adaptive divergence on the Z chromosome?

If natural selection has played a significant role in shaping variation and divergence of the Z-chromosome differently from autosomes, one may expect to find molecular footprints of selection events in the data. However, neither the HKA tests nor Tajima's *D* revealed any deviations from neutral expectance, neither at the Z-linked loci nor the autosomal ones.

Mank et al. (2010) analyzed genomic data from the chicken and the zebra finch and found evidence for an elevated d_N_/d_S_ ratio on the Z chromosome. They suggested that genetic drift might be an important contributing factor to this effect. Their argument was that the reduced effective population size of the Z chromosome, reinforced by a female-biased sex ratio due to sexual selection, would elevate the rate at which slightly deleterious nonsynonymous mutations becomes fixed by drift. An operational female-biased sex ratio (some males mate with more than one female) has been observed for both collared and pied flycatchers. In a study by Qvarnström et al. (2003), 4% of the collared flycatcher females were mated with an already mated male, while the corresponding figure in the pied flycatcher is around 10–15% (Lundberg and Alatalo 1992). No such studies have been done on the Atlas or semicollared flycatchers, but since they are closely related to the pied and collared flycatcher, it is likely that they have a similar mating system, and thus have a somewhat female-biased sex ratio.

Increased rate of genetic drift could explain (or contribute to explain) the observed reduction in polymorphism in our dataset. An elevated mutation rate on the Z-chromosome could in addition account for the elevated rate of divergence that we observe. A higher mutation rate is expected on the Z because mature sperm cells go through more cell divisions than egg cells. Hence, since the Z-chromosome spends two-third of its time in the male germ line, compared to the autosomes one-half, a male-biased mutation rate would elevate the overall mutation rate of the Z chromosome. Indeed, there is some evidence for a male-biased mutation rate in birds (e.g., Axelsson et al. 2004).

Although we acknowledge that genetic drift combined with an elevated mutation rate on the Z chromosome would be consistent with our results, we certainly do not rule out that hemizygous exposure of nonneutral alleles has contributed to the faster-Z effect. Indeed, in a methodologically similar study as the present one, on two of the species included here, and using a larger number of markers, Borge et al. (2005b) reported significant deviations from neutrality among the Z-linked markers according to an HKA test. The latter result is consistent with recurrent selective sweeps on the Z chromosome that would reduce variation within, and increase divergence between the taxa.

## Conclusions

To our knowledge, reduced variation coupled with an elevated rate of divergence on Z-linked loci relative to autosomal expectance has been found in all avian cases investigated so far. This consistency suggests that a common evolutionary force or set of forces related to peculiarities of the Z chromosome in itself shapes the pattern. We consider it unlikely that differential introgression is a sufficiently uniform evolutionary force to account for this seemingly general pattern, although it may be a contributing factor in certain cases (see e.g., Carling et al. 2010; Backström et al. 2010). Rather we suggest that the pattern is a manifestation of the faster-Z effect. Further studies are needed to evaluate the relative importance of elevated mutation rates, increased genetic drift, and more effective selection in shaping the Z chromosome differently from the other chromosomes.

## Supporting information

**Table S1.** Fixed and shared polymorphisms between species.

**Table S2.** Hudson-Kreitmann-Aguade tests.
