# Supplementary material for: Increased divergence but reduced variation on the Z chromosome relative to autosomes in Ficedula flycatchers: differential introgression or the faster-Z effect?
Source: Ecol Evol. 2012 Feb;2(2):379–96. doi: 10.1002/ece3.92 (PMC3298950; doi:10.1002/ece3.92)
Supplement: Supplementary file 1 [file ece30002-0379-SD1.doc]

**Table S1**. Fixed and shared polymorphisms between species. S-values are the number of variable sites that occur in or between species pairs. S_shared_ indicates the number of shared polymorphisms between the different species; S_fixed_ indicates the number of fixed differences between the species and S_average_ indicates the average pairwise difference between the species pairs. Total-Z is all Z-linked loci combined and total-A is all autosomal loci combined.

| Locus | S_P-Spa_ | S_C-It_ | S_shared_ | S_fixed_ | S_average_ | Length |
| --- | --- | --- | --- | --- | --- | --- |
| Aldob-6 | 3 | 1 | 0 | 2 | 3.61 | 437 |
| BRM-12 | 12 | 11 | 1 | 1 | 8.423 | 1437 |
| CHDZ | 3 | 1 | 0 | 1 | 1.227 | 638 |
| GHR | 4 | 4 | 1 | 1 | 1.674 | 555 |
| VLDLR | 3 | 12 | 0 | 1 | 4.514 | 562 |
| Total-Z | 25 | 29 | 2 | 6 | 19.448 | 3629 |
| ACLY-16 | 2 | 1 | 0 | 0 | 0.133 | 358 |
| ALAS1-8 | 6 | 9 | 0 | 0 | 2.678 | 290 |
| FAS-Y | 1 | 7 | 0 | 1 | 1.568 | 551 |
| RHO-1 | 7 | 6 | 0 | 1 | 4.333 | 371 |
| RPL30-3 | 14 | 21 | 9 | 0 | 5.582 | 983 |
| TGFB2-5 | 3 | 6 | 1 | 0 | 1.192 | 402 |
| Total-A | 33 | 50 | 10 | 2 | 15.486 | 2955 |
|  |  |  |  |  |  |  |
| Locus | S_P-Spa_ | S_S-Bul_ | S_shared_ | S_fixed_ | S_average_ | Length |
| Aldob-6 | 3 | 1 | 0 | 1 | 1.747 | 437 |
| BRM-12 | 12 | 23 | 2 | 2 | 8.453 | 1433 |
| CHDZ | 3 | 1 | 0 | 1 | 1.342 | 638 |
| GHR | 4 | 5 | 0 | 1 | 3.06 | 555 |
| VLDLR | 3 | 11 | 0 | 0 | 4.362 | 562 |
| Total-Z | 25 | 41 | 2 | 5 | 18.964 | 3625 |
| ACLY-16 | 2 | 1 | 0 | 0 | 0.133 | 358 |
| ALAS1-8 | 6 | 9 | 1 | 0 | 2.471 | 288 |
| FAS-Y | 1 | 6 | 0 | 0 | 0.396 | 551 |
| RHO-1 | 8 | 12 | 2 | 1 | 5.613 | 372 |
| RPL30-3 | 14 | 15 | 3 | 2 | 13.2 | 983 |
| TGFB2-5 | 3 | 6 | 0 | 0 | 2.231 | 401 |
| Total-A | 34 | 49 | 6 | 3 | 24.044 | 2953 |
|  |  |  |  |  |  |  |

| Locus | S_P-Spa_ | S_A-Mar_ | S_shared_ | S_fixed_ | S_average_ | Length |
| --- | --- | --- | --- | --- | --- | --- |
| Aldob-6 | 3 | 0 | 0 | 1 | 1.71 | 437 |
| BRM-12 | 12 | 20 | 2 | 2 | 10.324 | 1438 |
| CHDZ | 3 | 1 | 0 | 0 | 0.36 | 638 |
| GHR | 4 | 3 | 1 | 1 | 2.978 | 548 |
| VLDLR | 3 | 9 | 0 | 1 | 5.381 | 562 |
| Total-Z | 25 | 33 | 3 | 5 | 20.753 | 3623 |
| ACLY-16 | 2 | 0 | 0 | 0 | 0.1 | 358 |
| ALAS1-8 | 6 | 7 | 0 | 0 | 3.212 | 290 |
| FAS-Y | 1 | 0 | 0 | 0 | 0.029 | 551 |
| RHO-1 | 8 | 8 | 1 | 1 | 6.54 | 372 |
| RPL30-3 | 14 | 22 | 9 | 1 | 11.382 | 983 |
| TGFB2-5 | 3 | 6 | 0 | 0 | 1.831 | 402 |
| Total-A | 34 | 43 | 10 | 2 | 23.094 | 2956 |
|  |  |  |  |  |  |  |
| Locus | S_P-Spa_ | S_C-Hun_ | S_shared_ | S_fixed_ | S_average_ | Length |
| Aldob-6 | 3 | 1 | 0 | 2 | 3.616 | 437 |
| BRM-12 | 12 | 26 | 3 | 1 | 9.153 | 1438 |
| CHDZ | 3 | 4 | 0 | 1 | 1.394 | 638 |
| GHR | 4 | 4 | 1 | 1 | 1.658 | 555 |
| VLDLR | 3 | 13 | 0 | 1 | 4.621 | 558 |
| Total-Z | 25 | 48 | 4 | 6 | 20.442 | 3626 |
| ACLY-16 | 2 | 2 | 0 | 0 | 0.163 | 358 |
| ALAS1-8 | 6 | 9 | 0 | 0 | 2.537 | 290 |
| FAS-Y | 1 | 1 | 0 | 1 | 1.061 | 550 |
| RHO-1 | 8 | 12 | 1 | 1 | 4.812 | 371 |
| RPL30-3 | 14 | 23 | 4 | 0 | 6.208 | 982 |
| TGFB2-5 | 3 | 11 | 1 | 0 | 1.57 | 402 |
| Total-A | 34 | 58 | 6 | 2 | 16.351 | 2953 |
|  |  |  |  |  |  |  |
| Locus | S_P-Spa_ | S_P-Nor_ | S_shared_ | S_fixed_ | S_average_ | Length |
| Aldob-6 | 3 | 1 | 1 | 0 | 0.655 | 437 |
| BRM-12 | 12 | 23 | 6 | 0 | 4.958 | 1438 |
| CHDZ | 3 | 1 | 0 | 0 | 0.35 | 638 |
| GHR | 4 | 6 | 3 | 0 | 0.573 | 555 |
| VLDLR | 3 | 4 | 0 | 0 | 0.581 | 562 |
| Total-Z | 25 | 35 | 10 | 0 | 7.117 | 3630 |
| ACLY-16 | 2 | 0 | 0 | 0 | 0.1 | 358 |
| ALAS1-8 | 6 | 7 | 2 | 0 | 0.75 | 290 |
| FAS-Y | 1 | 2 | 0 | 0 | 0.329 | 551 |
| RHO-1 | 8 | 7 | 6 | 0 | 2.388 | 372 |
| RPL30-3 | 14 | 21 | 11 | 0 | 6.964 | 983 |
| TGFB2-5 | 3 | 7 | 3 | 0 | 1.046 | 402 |
| Total-A | 34 | 44 | 22 | 0 | 11.577 | 2956 |
|  |  |  |  |  |  |  |
| Locus | S_C-It_ | S_S-Bul_ | S_shared_ | S_fixed_ | S_average_ | Length |
| Aldob-6 | 1 | 1 | 0 | 3 | 3.937 | 437 |
| BRM-12 | 11 | 23 | 0 | 2 | 5.756 | 1433 |
| CHDZ | 1 | 1 | 0 | 0 | 0.181 | 638 |
| GHR | 4 | 5 | 0 | 0 | 1.663 | 555 |
| VLDLR | 12 | 11 | 3 | 0 | 2.969 | 562 |
| Total-Z | 29 | 41 | 3 | 5 | 14.506 | 3625 |
| ACLY-16 | 1 | 1 | 0 | 0 | 0.067 | 358 |
| ALAS1-8 | 9 | 9 | 1 | 0 | 4.084 | 288 |
| FAS-Y | 7 | 6 | 0 | 1 | 1.905 | 551 |
| RHO-1 | 6 | 11 | 4 | 0 | 3.733 | 371 |
| RPL30-3 | 21 | 15 | 8 | 3 | 12.405 | 983 |
| TGFB2-5 | 6 | 6 | 2 | 0 | 1.9 | 401 |
| Total-A | 50 | 48 | 15 | 4 | 24.094 | 2952 |
|  |  |  |  |  |  |  |
| Locus | S_C-It_ | S_A-Mar_ | S_shared_ | S_fixed_ | S_average_ | Length |
| Aldob-6 | 1 | 0 | 0 | 3 | 3.9 | 437 |
| BRM-12 | 11 | 20 | 1 | 1 | 8.898 | 1438 |
| CHDZ | 1 | 1 | 0 | 1 | 1.2 | 638 |
| GHR | 4 | 3 | 1 | 0 | 2.007 | 548 |
| VLDLR | 12 | 9 | 1 | 0 | 7.267 | 562 |
| Total-Z | 29 | 33 | 3 | 5 | 23.272 | 3623 |
| ACLY-16 | 1 | 0 | 0 | 0 | 0.033 | 358 |
| ALAS1-8 | 9 | 7 | 4 | 0 | 3.058 | 290 |
| FAS-Y | 7 | 0 | 0 | 1 | 1.538 | 551 |
| RHO-1 | 6 | 8 | 4 | 0 | 2.656 | 371 |
| RPL30-3 | 21 | 22 | 8 | 0 | 11.88 | 983 |
| TGFB2-5 | 6 | 6 | 0 | 0 | 1.533 | 402 |
| Total-A | 50 | 43 | 16 | 1 | 20.698 | 2955 |
|  |  |  |  |  |  |  |
| Locus | S_C-It_ | S_C-Hun_ | S_shared_ | S_fixed_ | S_average_ | Length |
| Aldob-6 | 1 | 1 | 1 | 0 | 0.175 | 437 |
| BRM-12 | 26 | 30 | 7 | 0 | 4.352 | 1438 |
| CHDZ | 1 | 4 | 0 | 0 | 0.233 | 638 |
| GHR | 4 | 4 | 1 | 0 | 0.744 | 555 |
| VLDLR | 10 | 13 | 8 | 0 | 2.898 | 558 |
| Total-Z | 42 | 52 | 17 | 0 | 8.402 | 3626 |
| ACLY-16 | 1 | 2 | 0 | 0 | 0.096 | 358 |
| ALAS1-8 | 9 | 9 | 7 | 0 | 1.669 | 290 |
| FAS-Y | 7 | 1 | 0 | 0 | 0.57 | 550 |
| RHO-1 | 6 | 11 | 4 | 0 | 1.8 | 370 |
| RPL30-3 | 21 | 23 | 9 | 0 | 4.915 | 982 |
| TGFB2-5 | 6 | 11 | 5 | 0 | 1.384 | 402 |
| Total-A | 50 | 57 | 25 | 0 | 10.434 | 2952 |
|  |  |  |  |  |  |  |
| Locus | S_C-It_ | S_P-Nor_ | S_shared_ | S_fixed_ | S_average_ | Length |
| Aldob-6 | 1 | 1 | 0 | 2 | 3.463 | 437 |
| BRM-12 | 11 | 23 | 2 | 1 | 9.451 | 1437 |
| CHDZ | 1 | 1 | 0 | 1 | 1.19 | 638 |
| GHR | 4 | 6 | 1 | 1 | 1.8 | 555 |
| VLDLR | 12 | 4 | 0 | 1 | 4.667 | 562 |
| Total-Z | 29 | 35 | 3 | 6 | 20.571 | 3629 |
| ACLY-16 | 0 | 1 | 0 | 0 | 0.033 | 358 |
| ALAS1-8 | 9 | 7 | 2 | 0 | 2.515 | 290 |
| FAS-Y | 7 | 2 | 0 | 1 | 1.838 | 551 |
| RHO-1 | 6 | 6 | 0 | 1 | 4.096 | 371 |
| RPL30-3 | 21 | 21 | 10 | 0 | 9.586 | 983 |
| TGFB2-5 | 6 | 7 | 1 | 0 | 1.634 | 402 |
| Total-A | 49 | 44 | 13 | 2 | 19.702 | 2955 |
|  |  |  |  |  |  |  |
| Locus | S_S-Bul_ | S_A-Mar_ | S_shared_ | S_fixed_ | S_average_ | Length |
| Aldob-6 | 1 | 0 | 0 | 2 | 2.037 | 437 |
| BRM-12 | 23 | 20 | 2 | 0 | 8.367 | 1434 |
| CHDZ | 1 | 1 | 0 | 1 | 1.315 | 638 |
| GHR | 5 | 3 | 0 | 0 | 3.03 | 548 |
| VLDLR | 11 | 9 | 1 | 0 | 7.068 | 562 |
| Total-Z | 41 | 33 | 3 | 3 | 21.817 | 3619 |
| ACLY-16 | 1 | 0 | 0 | 0 | 0.033 | 358 |
| ALAS1-8 | 9 | 7 | 1 | 0 | 4.609 | 288 |
| FAS-Y | 6 | 0 | 0 | 0 | 0.367 | 551 |
| RHO-1 | 12 | 8 | 4 | 0 | 4.493 | 372 |
| RPL30-3 | 15 | 22 | 5 | 1 | 10.797 | 983 |
| TGFB2-5 | 6 | 6 | 0 | 0 | 2.533 | 401 |
| Total-A | 49 | 43 | 10 | 1 | 22.832 | 2953 |
|  |  |  |  |  |  |  |
| Locus | S_S-Bul_ | S_C-Hun_ | S_shared_ | S_fixed_ | S_average_ | Length |
| Aldob-6 | 1 | 1 | 0 | 3 | 3.943 | 437 |
| BRM-12 | 23 | 26 | 3 | 0 | 6.757 | 1434 |
| CHDZ | 1 | 4 | 0 | 0 | 0.348 | 638 |
| GHR | 5 | 4 | 0 | 0 | 1.869 | 555 |
| VLDLR | 11 | 13 | 3 | 0 | 2.404 | 558 |
| Total-Z | 41 | 48 | 6 | 3 | 15.321 | 3622 |
| ACLY-16 | 1 | 2 | 0 | 0 | 0.096 | 358 |
| ALAS1-8 | 9 | 9 | 1 | 0 | 3.967 | 288 |
| FAS-Y | 6 | 1 | 0 | 1 | 1.398 | 550 |
| RHO-1 | 12 | 12 | 4 | 0 | 4.448 | 371 |
| RPL30-3 | 15 | 23 | 3 | 5 | 12.865 | 982 |
| TGFB2-5 | 6 | 11 | 3 | 0 | 2.438 | 401 |
| Total-A | 49 | 58 | 11 | 6 | 25.212 | 2950 |
|  |  |  |  |  |  |  |
| Locus | S_S-Bul_ | S_P-Nor_ | S_shared_ | S_fixed_ | S_average_ | Length |
| Aldob-6 | 1 | 1 | 0 | 1 | 1.6 | 437 |
| BRM-12 | 23 | 23 | 3 | 3 | 9.486 | 1433 |
| CHDZ | 1 | 1 | 0 | 1 | 1.304 | 638 |
| GHR | 5 | 6 | 0 | 1 | 3.15 | 555 |
| VLDLR | 11 | 4 | 0 | 0 | 4.515 | 562 |
| Total-Z | 41 | 35 | 3 | 6 | 20.055 | 3625 |
| ACLY-16 | 1 | 0 | 0 | 0 | 0.033 | 358 |
| ALAS1-8 | 9 | 7 | 0 | 0 | 2.41 | 288 |
| FAS-Y | 6 | 2 | 0 | 0 | 0.667 | 551 |
| RHO-1 | 12 | 7 | 2 | 1 | 5.538 | 372 |
| RPL30-3 | 15 | 21 | 4 | 0 | 12.516 | 983 |
| TGFB2-5 | 6 | 7 | 1 | 0 | 2.656 | 401 |
| Total-A | 49 | 44 | 7 | 1 | 23.82 | 2953 |
|  |  |  |  |  |  |  |
| Locus | S_A-Mar_ | S_C-Hun_ | S_shared_ | S_fixed_ | S_average_ | Length |
| Aldob-6 | 0 | 1 | 0 | 3 | 3.906 | 437 |
| BRM-12 | 20 | 26 | 2 | 0 | 8.926 | 1439 |
| CHDZ | 1 | 4 | 0 | 1 | 1.367 | 638 |
| GHR | 3 | 4 | 1 | 0 | 2.063 | 548 |
| VLDLR | 8 | 13 | 1 | 0 | 6.39 | 558 |
| Total-Z | 32 | 48 | 4 | 4 | 22.652 | 3620 |
| ACLY-16 | 0 | 2 | 0 | 0 | 0.063 | 358 |
| ALAS1-8 | 7 | 9 | 3 | 0 | 3.238 | 290 |
| FAS-Y | 0 | 1 | 0 | 1 | 1.031 | 550 |
| RHO-1 | 8 | 12 | 4 | 0 | 3.322 | 371 |
| RPL30-3 | 22 | 23 | 3 | 3 | 12.567 | 982 |
| TGFB2-5 | 6 | 11 | 0 | 0 | 2.129 | 402 |
| Total-A | 43 | 58 | 10 | 4 | 22.35 | 2953 |
|  |  |  |  |  |  |  |
| Locus | S_A-Mar_ | S_P-Nor_ | S_shared_ | S_fixed_ | S_average_ | Length |
| Aldob-6 | 0 | 1 | 0 | 1 | 1.563 | 437 |
| BRM-12 | 20 | 23 | 3 | 3 | 11.558 | 1438 |
| CHDZ | 1 | 1 | 0 | 0 | 0.323 | 638 |
| GHR | 3 | 6 | 1 | 1 | 3.106 | 548 |
| VLDLR | 9 | 4 | 0 | 1 | 5.533 | 562 |
| Total-Z | 33 | 35 | 4 | 6 | 22.083 | 3623 |
| ACLY-16 | 0 | 0 | 0 | 0 | 0 | 358 |
| ALAS1-8 | 7 | 7 | 2 | 0 | 3.081 | 290 |
| FAS-Y | 0 | 2 | 0 | 0 | 0.3 | 551 |
| RHO-1 | 8 | 7 | 1 | 1 | 6.515 | 372 |
| RPL30-3 | 22 | 21 | 12 | 0 | 10.19 | 983 |
| TGFB2-5 | 6 | 7 | 0 | 0 | 2.285 | 402 |
| Total-A | 43 | 44 | 15 | 1 | 22.371 | 2956 |
|  |  |  |  |  |  |  |
| Locus | S_C-Hun_ | S_P-Nor_ | S_shared_ | S_fixed_ | S_average_ | Length |
| Aldob-6 | 1 | 1 | 0 | 2 | 3.469 | 437 |
| BRM-12 | 20 | 23 | 3 | 3 | 11.558 | 1438 |
| CHDZ | 4 | 1 | 0 | 1 | 1.356 | 638 |
| GHR | 4 | 6 | 1 | 1 | 1.791 | 555 |
| VLDLR | 13 | 4 | 0 | 1 | 4.773 | 558 |
| Total-Z | 42 | 35 | 4 | 8 | 22.947 | 3626 |
| ACLY-16 | 2 | 0 | 0 | 0 | 0.063 | 358 |
| ALAS1-8 | 9 | 7 | 1 | 0 | 2.383 | 290 |
| FAS-Y | 1 | 2 | 0 | 1 | 1.331 | 550 |
| RHO-1 | 12 | 7 | 1 | 1 | 4.568 | 371 |
| RPL30-3 | 23 | 21 | 3 | 0 | 10.288 | 982 |
| TGFB2-5 | 11 | 7 | 2 | 0 | 1.948 | 402 |
| Total-A | 58 | 44 | 7 | 2 | 20.581 | 2953 |

**Table S2**. Hudson-Kreitmann-Aguade tests. Expected and observed number of segregating sites for each species, and the divergence (average no. of nucleotide differences) between the different species pairs.

|  | A-Mar |  | C-It |  | Divergence | |
| --- | --- | --- | --- | --- | --- | --- |
| Locus | Observed | Expected | Observed | Expected | Observed | Expected |
| ALDOB_6 | 0 | 1.95 | 1 | 1.84 | 3.9 | 1.11 |
| BRM_12 | 20 | 15.84 | 11 | 14.88 | 8.9 | 9.17 |
| CHDZ | 1 | 1.28 | 1 | 1.2 | 1.2 | 0.73 |
| GHR | 3 | 3.57 | 4 | 3.4 | 2.01 | 2.03 |
| VLDLR | 9 | 11.26 | 12 | 10.59 | 7.27 | 6.41 |
| ACLY_16 | 0 | 0.43 | 1 | 0.4 | 0.03 | 0.21 |
| ALAS1_8 | 7 | 7.85 | 9 | 7.38 | 3.06 | 3.83 |
| FAS_Y | 0 | 3.49 | 7 | 3.28 | 1.54 | 1.77 |
| RHO_1 | 8 | 6.86 | 6 | 6.45 | 2.66 | 3.34 |
| RPL30_3 | 30 | 25.90 | 21 | 24.34 | 11.88 | 12.64 |
| TGFB2_5 | 6 | 5.57 | 6 | 5.24 | 1.53 | 2.72 |
|  |  |  |  |  |  | P= 0.61 |
|  |  |  |  |  |  |  |
|  | A-Mar |  | P-Spa |  | Divergence | |
| Locus | Observed | Expected | Observed | Expected | Observed | Expected |
| ALDOB_6 | 0 | 1.92 | 3 | 1.53 | 1.71 | 1.23 |
| BRM_12 | 20 | 17.69 | 12 | 13.24 | 10.32 | 11.39 |
| CHDZ | 1 | 1.80 | 3 | 1.42 | 0.36 | 1.14 |
| GHR | 3 | 4.14 | 4 | 3.26 | 2.98 | 2.58 |
| VLDLR | 9 | 7.25 | 3 | 5.55 | 5.38 | 4.58 |
| ACLY_16 | 0 | 0.91 | 2 | 0.71 | 0.1 | 0.48 |
| ALAS1_8 | 7 | 6.94 | 6 | 5.59 | 3.21 | 3.68 |
| FAS_Y | 0 | 0.43 | 1 | 0.36 | 0.03 | 0.24 |
| RHO_1 | 8 | 9.75 | 8 | 7.61 | 6.54 | 5.18 |
| RPL30_3 | 22 | 20.50 | 14 | 16 | 11.38 | 10.88 |
| TGFB2_5 | 6 | 4.64 | 3 | 3.73 | 1.83 | 2.46 |
|  |  |  |  |  |  | P= 0.88 |
|  |  |  |  |  |  |  |
|  | A-Mar |  | C-Hun |  | Divergence | |
| Locus | Observed | Expected | Observed | Expected | Observed | Expected |
| ALDOB_6 | 0 | 1.63 | 1 | 2.23 | 3.91 | 1.05 |
| BRM_12 | 20 | 18.13 | 26 | 24.83 | 8.93 | 11.97 |
| CHDZ | 1 | 1.12 | 1 | 1.51 | 1.37 | 0.73 |
| GHR | 3 | 2.99 | 4 | 4.14 | 2.06 | 1.94 |
| VLDLR | 8 | 9.12 | 13 | 12.39 | 6.39 | 5.87 |
| ACLY_16 | 0 | 0.70 | 2 | 0.96 | 0.06 | 0.39 |
| ALAS1_8 | 7 | 6.57 | 9 | 8.99 | 3.24 | 3.68 |
| FAS_Y | 0 | 0.68 | 1 | 0.96 | 1.03 | 0.39 |
| RHO_1 | 8 | 7.86 | 11 | 10.16 | 3.41 | 4.39 |
| RPL30_3 | 22 | 19.67 | 23 | 26.88 | 12.57 | 11.01 |
| TGFB2_5 | 6 | 6.53 | 11 | 8.94 | 2.13 | 3.66 |
|  |  |  |  |  |  | P= 0.84 |
|  |  |  |  |  |  |  |
|  | A-Mar |  | P-Nor |  | Divergence | |
| Locus | Observed | Expected | Observed | Expected | Observed | Expected |
| ALDOB_6 | 0 | 0.95 | 1 | 1.01 | 1.56 | 0.6 |
| BRM_12 | 20 | 20.46 | 23 | 20.9 | 11.56 | 13.2 |
| CHDZ | 1 | 0.86 | 1 | 0.91 | 0.32 | 0.55 |
| GHR | 3 | 4.47 | 6 | 4.8 | 3.11 | 2.84 |
| VLDLR | 9 | 6.92 | 4 | 7.22 | 5.53 | 4.39 |
| ACLY_16 | 0 | 0.00 | 0 | 0 | 0 | 0 |
| ALAS1_8 | 7 | 6.57 | 7 | 6.96 | 3.08 | 3.55 |
| FAS_Y | 0 | 0.87 | 2 | 0.94 | 0.3 | 0.49 |
| RHO_1 | 8 | 8.27 | 7 | 8.77 | 6.51 | 4.47 |
| RPL30_3 | 22 | 20.74 | 21 | 21.25 | 10.19 | 11.2 |
| TGFB2_5 | 6 | 5.88 | 7 | 6.23 | 2.29 | 3.17 |
|  |  |  |  |  |  | P= 0.99 |
|  |  |  |  |  |  |  |
|  | A-Mar |  | S-Bul |  | Divergence | |
| Locus | Observed | Expected | Observed | Expected | Observed | Expected |
| ALDOB_6 | 0 | 1.15 | 1 | 1.23 | 2.53 | 2.78 |
| BRM_12 | 20 | 19.45 | 23 | 20.6 | 2.04 | 0.66 |
| CHDZ | 1 | 1.26 | 1 | 1.34 | 8.37 | 11.32 |
| GHR | 3 | 4.16 | 5 | 4.49 | 1.31 | 0.72 |
| VLDLR | 9 | 10.26 | 11 | 10.93 | 7.07 | 5.88 |
| ACLY_16 | 0 | 0.40 | 1 | 0.44 | 0.03 | 0.2 |
| ALAS1_8 | 7 | 8.69 | 7 | 7.99 | 0.03 | 0.2 |
| FAS_Y | 0 | 2.40 | 6 | 2.73 | 4.61 | 3.94 |
| RHO_1 | 8 | 9.45 | 12 | 10.35 | 0.37 | 1.24 |
| RPL30_3 | 30 | 21.87 | 15 | 12.07 | 4.49 | 4.69 |
| TGFB2_5 | 6 | 5.62 | 6 | 6.14 | 10.8 | 10.85 |
|  |  |  |  |  |  | P= 0.84 |
|  |  |  |  |  |  |  |
|  | C-It |  | P-Spa |  | Divergence | |
| Locus | Observed | Expected | Observed | Expected | Observed | Expected |
| ALDOB_6 | 1 | 3.43 | 3 | 2.54 | 3.61 | 1.64 |
| BRM_12 | 11 | 14.35 | 12 | 10.11 | 8.42 | 6.97 |
| CHDZ | 1 | 2.35 | 3 | 1.75 | 1.23 | 1.13 |
| GHR | 4 | 4.36 | 4 | 3.23 | 1.67 | 2.08 |
| VLDLR | 12 | 8.88 | 3 | 6.4 | 4.51 | 4.24 |
| ACLY_16 | 1 | 1.46 | 2 | 1.07 | 0.13 | 0.6 |
| ALAS1_8 | 9 | 8.15 | 6 | 6.17 | 2.68 | 3.36 |
| FAS_Y | 7 | 4.34 | 1 | 3.38 | 1.57 | 1.85 |
| RHO_1 | 6 | 8.08 | 7 | 5.93 | 4.33 | 3.33 |
| RPL30_3 | 21 | 18.91 | 14 | 13.87 | 5.58 | 7.8 |
| TGFB2_5 | 6 | 4.70 | 3 | 3.56 | 1.19 | 1.94 |
|  |  |  |  |  |  | P= 0.92 |

|  | C-It |  | C-Hun |  | Divergence | |
| --- | --- | --- | --- | --- | --- | --- |
| Locus | Observed | Expected | Observed | Expected | Observed | Expected |
| ALDOB_6 | 1 | 0.84 | 1 | 1.14 | 0.17 | 0.25 |
| BRM_12 | 11 | 15.91 | 26 | 21.64 | 4.35 | 4.77 |
| CHDZ | 1 | 2.04 | 4 | 2.72 | 0.23 | 0.6 |
| GHR | 4 | 3.37 | 4 | 4.58 | 0.74 | 0.99 |
| VLDLR | 10 | 10.04 | 13 | 13.53 | 2.9 | 2.94 |
| ACLY_16 | 1 | 1.20 | 2 | 1.6 | 0.1 | 0.35 |
| ALAS1_8 | 9 | 7.55 | 9 | 10.24 | 1.67 | 2.22 |
| FAS_Y | 7 | 3.22 | 1 | 4.52 | 0.57 | 0.98 |
| RHO_1 | 6 | 7.01 | 10 | 9 | 1.75 | 2.06 |
| RPL30_3 | 21 | 18.78 | 23 | 25.46 | 4.92 | 5.53 |
| TGFB2_5 | 6 | 7.05 | 11 | 9.57 | 1.38 | 2.08 |
|  |  |  |  |  |  | P= 0.99 |
|  |  |  |  |  |  |  |
|  | C-It |  | P-Nor |  | Divergence | |
| Locus | Observed | Expected | Observed | Expected | Observed | Expected |
| ALDOB_6 | 1 | 2.14 | 1 | 2.15 | 3.46 | 1.18 |
| BRM_12 | 11 | 17.18 | 23 | 16.64 | 9.45 | 9.62 |
| CHDZ | 1 | 1.25 | 1 | 1.25 | 1.19 | 0.69 |
| GHR | 4 | 4.62 | 4 | 4.64 | 1.8 | 2.54 |
| VLDLR | 12 | 8.14 | 4 | 8.05 | 4.67 | 4.48 |
| ACLY_16 | 1 | 0.42 | 0 | 0.42 | 0.03 | 0.2 |
| ALAS1_8 | 9 | 7.47 | 7 | 7.5 | 2.52 | 3.55 |
| FAS_Y | 7 | 4.31 | 2 | 4.41 | 1.84 | 2.12 |
| RHO_1 | 6 | 6.49 | 6 | 6.52 | 4.1 | 3.08 |
| RPL30_3 | 21 | 21.09 | 21 | 20.47 | 9.59 | 10.02 |
| TGFB2_5 | 6 | 5.90 | 7 | 5.93 | 1.63 | 2.8 |
|  |  |  |  |  |  | P= 0.85 |
|  |  |  |  |  |  |  |
|  | C-It |  | S-Bul |  | Divergence | |
| Locus | Observed | Expected | Observed | Expected | Observed | Expected |
| ALDOB_6 | 1 | 2.25 | 1 | 2.51 | 3.94 | 1.18 |
| BRM_12 | 11 | 15.04 | 23 | 16.72 | 5.76 | 8 |
| CHDZ | 1 | 0.83 | 1 | 0.92 | 0.18 | 0.43 |
| GHR | 4 | 4.04 | 5 | 4.51 | 1.66 | 2.12 |
| VLDLR | 12 | 9.83 | 11 | 10.99 | 3.97 | 5.15 |
| ACLY_16 | 1 | 0.79 | 1 | 0.91 | 0.07 | 0.37 |
| ALAS1_8 | 9 | 8.50 | 9 | 9.69 | 4.08 | 3.89 |
| FAS_Y | 7 | 5.59 | 6 | 6.65 | 1.91 | 2.67 |
| RHO_1 | 6 | 7.95 | 11 | 9.13 | 3.73 | 3.66 |
| RPL30_3 | 21 | 18.86 | 15 | 20.83 | 12.4 | 8.69 |
| TGFB2_5 | 6 | 5.34 | 6 | 6.11 | 1.9 | 2.45 |
|  |  |  |  |  |  | P= 0.95 |

|  |  |  |  |  |  |  |
| --- | --- | --- | --- | --- | --- | --- |
|  | P-Spa |  | C-Hun |  | Divergence | |
| Locus | Observed | Expected | Observed | Expected | Observed | Expected |
| ALDOB_6 | 3 | 2.35 | 1 | 3.84 | 3.62 | 1.43 |
| BRM_12 | 12 | 13.99 | 26 | 24.08 | 9.15 | 9.08 |
| CHDZ | 3 | 2.61 | 4 | 4.2 | 1.39 | 1.58 |
| GHR | 4 | 2.98 | 4 | 4.87 | 1.66 | 1.81 |
| VLDLR | 0 | 0.00 | 0 | 0 | 0 | 0 |
| ACLY_16 | 2 | 1.30 | 2 | 2.15 | 0.16 | 0.71 |
| ALAS1_8 | 6 | 5.61 | 9 | 8.98 | 2.54 | 2.95 |
| FAS_Y | 1 | 0.97 | 1 | 1.57 | 1.06 | 0.52 |
| RHO_1 | 8 | 7.68 | 11 | 11.98 | 4.8 | 4.15 |
| RPL30_3 | 14 | 13.53 | 23 | 22.35 | 6.21 | 7.33 |
| TGFB2_5 | 3 | 4.98 | 11 | 7.97 | 1.57 | 2.62 |
|  |  |  |  |  |  | P= 0.99 |
|  |  |  |  |  |  |  |
|  | P-Spa |  | P-Nor |  | Divergence | |
| Locus | Observed | Expected | Observed | Expected | Observed | Expected |
| ALDOB_6 | 3 | 1.75 | 1 | 2.36 | 0.66 | 0.55 |
| BRM_12 | 12 | 14.79 | 23 | 20.2 | 4.96 | 4.97 |
| CHDZ | 3 | 1.63 | 1 | 2.2 | 0.35 | 0.51 |
| GHR | 4 | 3.97 | 6 | 5.35 | 0.57 | 1.25 |
| VLDLR | 3 | 2.82 | 4 | 3.84 | 0.58 | 0.91 |
| ACLY_16 | 2 | 0.79 | 0 | 1.07 | 0.1 | 0.24 |
| ALAS1_8 | 6 | 5.25 | 7 | 6.91 | 0.75 | 1.58 |
| FAS_Y | 1 | 1.28 | 2 | 1.66 | 0.33 | 0.39 |
| RHO_1 | 8 | 6.51 | 1 | 8.85 | 2.39 | 2.03 |
| RPL30_3 | 14 | 15.99 | 21 | 21 | 6.96 | 4.98 |
| TGFB2_5 | 3 | 4.22 | 7 | 5.56 | 1.05 | 1.27 |
|  |  |  |  |  |  | P= 0.99 |
|  |  |  |  |  |  |  |
|  | P-Spa |  | S-Bul |  | Divergence | |
| Locus | Observed | Expected | Observed | Expected | Observed | Expected |
| ALDOB_6 | 3 | 1.75 | 1 | 2.62 | 1.75 | 1.37 |
| BRM_12 | 12 | 12.78 | 23 | 20.01 | 8.45 | 10.66 |
| CHDZ | 3 | 1.63 | 1 | 2.44 | 1.34 | 1.27 |
| GHR | 4 | 3.67 | 5 | 5.51 | 3.06 | 2.88 |
| VLDLR | 3 | 5.49 | 11 | 8.45 | 4.36 | 4.42 |
| ACLY_16 | 2 | 0.97 | 1 | 1.51 | 0.13 | 0.65 |
| ALAS1_8 | 6 | 5.56 | 9 | 8.31 | 2.47 | 3.6 |
| FAS_Y | 1 | 2.32 | 6 | 3.54 | 0.4 | 1.53 |
| RHO_1 | 8 | 7.94 | 12 | 12.33 | 5.61 | 5.34 |
| RPL30_3 | 14 | 13.32 | 15 | 19.92 | 13.2 | 8.96 |
| TGFB2_5 | 3 | 3.56 | 6 | 5.35 | 2.23 | 2.32 |
|  |  |  |  |  |  | P= 0.96 |

|  |  |  |  |  |  |  |
| --- | --- | --- | --- | --- | --- | --- |
|  | C-Hun |  | P-Nor |  | Divergence | |
| Locus | Observed | Expected | Observed | Expected | Observed | Expected |
| ALDOB_6 | 1 | 2.50 | 1 | 1.9 | 3.47 | 1.06 |
| BRM_12 | 26 | 27.35 | 23 | 20.07 | 10.26 | 11.84 |
| CHDZ | 4 | 2.88 | 1 | 2.26 | 1.36 | 1.25 |
| GHR | 4 | 5.39 | 6 | 4.11 | 1.79 | 2.3 |
| VLDLR | 13 | 9.98 | 4 | 7.54 | 4.77 | 4.25 |
| ACLY_16 | 2 | 0.97 | 0 | 0.74 | 0.06 | 0.36 |
| ALAS1_8 | 9 | 8.60 | 7 | 6.56 | 2.38 | 3.22 |
| FAS_Y | 1 | 2.04 | 2 | 1.53 | 1.33 | 0.76 |
| RHO_1 | 11 | 10.24 | 7 | 8.26 | 4.54 | 4.04 |
| RPL30_3 | 23 | 25.71 | 21 | 18.95 | 10.29 | 9.62 |
| TGFB2_5 | 11 | 9.34 | 7 | 7.12 | 1.95 | 3.49 |
|  |  |  |  |  |  | P= 0.90 |
|  |  |  |  |  |  |  |
|  | C-Hun |  | S-Bul |  | Divergence | |
| Locus | Observed | Expected | Observed | Expected | Observed | Expected |
| ALDOB_6 | 1 | 2.65 | 1 | 2.24 | 3.94 | 1.5 |
| BRM_12 | 26 | 24.85 | 23 | 20.88 | 6.76 | 10.02 |
| CHDZ | 4 | 2.36 | 1 | 2.03 | 0.5 | 0.96 |
| GHR | 4 | 4.46 | 5 | 3.77 | 1 | 1.77 |
| VLDLR | 13 | 11.73 | 11 | 10 | 2.4 | 4.67 |
| ACLY_16 | 2 | 1.39 | 1 | 1.21 | 0.1 | 0.5 |
| ALAS1_8 | 9 | 9.90 | 9 | 8.55 | 3.97 | 3.51 |
| FAS_Y | 1 | 3.77 | 6 | 3.28 | 1.4 | 1.35 |
| RHO_1 | 11 | 11.99 | 12 | 11.01 | 4.51 | 4.51 |
| RPL30_3 | 23 | 23.16 | 15 | 19.43 | 12.87 | 8.27 |
| TGFB2_5 | 11 | 8.74 | 6 | 7.59 | 2.44 | 3.11 |
|  |  |  |  |  |  | P= 0.75 |
|  |  |  |  |  |  |  |
|  | P-Nor |  | S-Bul |  | Divergence | |
| Locus | Observed | Expected | Observed | Expected | Observed | Expected |
| ALDOB_6 | 1 | 1.29 | 1 | 1.49 | 1.6 | 0.82 |
| BRM_12 | 23 | 19.81 | 24 | 23.53 | 9.49 | 13.15 |
| CHDZ | 1 | 1.19 | 1 | 1.37 | 1.3 | 0.75 |
| GHR | 4 | 4.37 | 5 | 5.03 | 3.15 | 2.76 |
| VLDLR | 4 | 7.58 | 11 | 9.03 | 6.39 | 4.78 |
| ACLY_16 | 0 | 0.38 | 1 | 0.45 | 0.03 | 0.21 |
| ALAS1_8 | 7 | 6.78 | 9 | 7.98 | 2.41 | 3.65 |
| FAS_Y | 2 | 3.15 | 6 | 3.79 | 0.67 | 1.73 |
| RHO_1 | 7 | 9.00 | 12 | 10.66 | 5.54 | 4.88 |
| RPL30_3 | 21 | 17.70 | 15 | 20.89 | 12.52 | 9.92 |
| TGFB2_5 | 7 | 5.57 | 6 | 6.79 | 2.66 | 3.11 |
|  |  |  |  |  |  | P= 0.99 |
|  |  |  |  |  |  |  |

A-Mar = Atlas from Morocco, C-It = collared from Italy, P-Spa = pied from Spain, C-Hun = collared from Hungary, P-Nor = pied from Norway, S-Bul = semicollared from Bulgaria
